# Emu Oil Reduces Small Intestinal Inflammation in the Absence of Clinical Improvement in a Rat Model of Indomethacin-Induced Enteropathy

**DOI:** 10.1155/2013/429706

**Published:** 2013-03-14

**Authors:** Suzanne M. Abimosleh, Cuong D. Tran, Gordon S. Howarth

**Affiliations:** ^1^Department of Gastroenterology, Women's and Children's Hospital, North Adelaide, SA 5006, Australia; ^2^Discipline of Physiology, School of Medical Sciences, Faculty of Health Sciences, The University of Adelaide, Adelaide, SA 5005, Australia; ^3^School of Animal and Veterinary Sciences, The University of Adelaide, Roseworthy Campus, Roseworthy, SA 5371, Australia

## Abstract

Nonsteroidal-anti-inflammatory-drug (NSAID) enteropathy is characterized by small intestinal damage and ulceration. Emu Oil (EO) has previously been reported to reduce intestinal inflammation. *Aim*. We investigated EO for its potential to attenuate NSAID-enteropathy in rats. *Methods*. Male Sprague Dawley rats (*n* = 10/group) were gavaged with Water, Olive Oil (OO), or EO (0.5 mL; days 0–12) and with 0.5 mL Water or the NSAID, Indomethacin (8 mg/kg; days 5–12) daily. Disease activity index (DAI), 13C-sucrose breath test (SBT), organ weights, intestinal damage severity (IDS), and myeloperoxidase (MPO) activity were assessed. *P* < 0.05 was considered significant. *Results*. In Indomethacin-treated rats, DAI was elevated (days 10–12) and SBT values (56%) and thymus weight (55%) were decreased, relative to normal controls. Indomethacin increased duodenum (68%), colon (24%), SI (48%), caecum (48%), liver (51%) and spleen (88%) weights, IDS scores, and MPO levels (jejunum: 195%, ileum: 104%) compared to normal controls. Jejunal MPO levels were decreased (64%) by both EO and OO, although only EO decreased ileal MPO (50%), compared to Indomethacin controls. *Conclusions*. EO reduced acute intestinal inflammation, whereas other parameters of Indomethacin-induced intestinal injury were not affected significantly. Increased EO dose and/or frequency of administration could potentially improve clinical efficacy.

## 1. Introduction

Nonsteroidal anti-inflammatory drugs (NSAIDs) are among the most widely prescribed pharmaceutical agents, with approximately 30 million patients worldwide ingesting NSAIDs daily [1, 2]. NSAIDs have been indicated as an effective treatment option for rheumatic and musculoskeletal conditions [3] and to potentially lower the risk of cardiovascular and cerebrovascular insults. Indeed, recent studies have indicated efficacy for the treatment of colon cancer [2, 4]. NSAIDs represent a highly effective class of drug; however, their use is often associated with a broad spectrum of adverse effects, predominantly within the gastrointestinal (GI) tract [2, 3]. The adverse events in the stomach, duodenum, jejunum, and ileum are collectively termed NSAID-gastroenteropathy [5].

NSAID-associated intestinal toxicity has several manifestations including increased mucosal permeability, inflammation and ulceration, and in severe cases, bowel perforation [5]. Clinically evident features include anaemia, bleeding, mucosal diaphragms, strictures, and chronic bowel inflammation [6]. Serious injury to the small intestine (SI) has been estimated to account for one-third of all NSAID-associated complications [6]. 

The pathogenesis of NSAID-enteropathy is proposed to commence via a direct intestinal insult together with its enterohepatic and consequent systemic effects [3, 5, 7]. Entrance of the acidic NSAID into enterocytes induces severe stress in the endoplasmic reticulum and mitochondria leading to cell death [3, 8]. An increase in mucosal permeability follows, which facilitates the translocation and action of luminal factors such as bile acids, dietary macromolecules, components of pancreatic juice, and bacteria, thereby activating the inflammatory cascade [8, 9]. Within the mucosa, tumor necrosis factor-*α* (TNF-*α*) expression promotes autoregulation and expression of other proinflammatory cytokines including interleukin-1*β* (IL-1*β*) and IL-6 [10]. Furthermore, neutrophils are recruited and infiltrated at the area of ulceration [3, 5, 10]. 

Commonly prescribed NSAIDs include Aspirin, Ibuprofen, Naproxen, Ketoprofen, and Indomethacin [4]. The enzyme, cyclooxygenase (COX), which exists as two isoforms, is the target for NSAID action. COX converts arachidonic acid (AA) to prostaglandin G_2 _(PGG_2_), the common precursor of major prostanoids which include PGD_2_, PGE_2_, prostacyclin (PGI_2_), and thromboxane (TXA_2_) [11]. The two isoforms of COX generally have distinct functions; COX-1-derived PGs maintain mucosal integrity whereas COX-2 is rapidly induced in response to proinflammatory stimuli leading to PG production at inflammatory sites [12]. 

Historically, GI injury has been primarily associated with nonselective COX-1/2 inhibitors. On the basis of these findings, selective COX-2 inhibitors including Celecoxib and Meloxicam were developed. These were initially reported to elicit analgesic properties accompanied by minimal GI inflammatory side effects [13]. However, a number of recent reports have suggested that the incidence of jejunal and ileal injury induced by chronic use of selective COX-2 inhibitors may be as high as nonselective therapies [5, 14–16]. The development of new therapies is therefore imperative to ameliorate the side effects of NSAID use, particularly in patients undergoing long-term NSAID-treatment.

Favorable effects of diets high in n-3 polyunsaturated fatty acids (PUFAs) have been well documented in inflammatory conditions including rheumatoid arthritis [17] and inflammatory bowel disease (IBD) [18]. Cell culture studies with n-3 PUFAs have shown inhibition of COX-2 production and proinflammatory cytokines including TNF-*α*, interleukin-1 (IL-1), IL-6, IL-8, and IL-12 [19–21]. Furthermore, animal studies with marine oils rich in n-3 PUFAs support the *in vitro* observations. For example, Fish Oil reduced levels of TNF-*α*, IL-12, and IL-1*β* in a mouse model of IBD [22]. Recently, attention has been directed towards animal-derived oils with high levels of PUFAs, such as the oil derived from the Australian ratite bird, the Emu *(Dromaius novaehollandiae)* [23, 24]. 

Indigenous Australians first used Emu Oil to facilitate wound healing, pain alleviation and treatment of inflamed joints [23, 25, 26]. Topical application of Emu Oil decreased levels of TNF-*α* and IL-1*α* in a mouse model of adjuvant-induced inflammation [27]. These cytokines are involved in the pathogenesis of NSAID-enteropathy [5]. Abimosleh et al. [24] reported that orally administered Emu Oil improved selected parameters associated with the manifestation of experimental IBD in rats. Furthermore, Lindsay et al. [28] demonstrated that Emu Oil reduced ileal myeloperoxidase activity indicative of neutrophil infiltration and improved mucosal architecture in a rat model of intestinal mucositis.

Therefore, we hypothesized that orally administered Emu Oil would protect against Indomethacin-induced enteropathy in rats.

## 2. Materials and Methods

### 2.1. General Experimental Procedures

#### 2.1.1. Animal Studies

Throughout acclimatization and the experimental period, male Sprague Dawley rats (140–170 g) were individually housed in metabolism cages (Tecniplast, PA, USA) at room temperature with a light:dark cycle of 12 hours. All rats were given *ad libitum *access to food (standard 18% casein-based diet [29]) and water, and were acclimatized for 2 days prior to experimentation. All animal studies were conducted in compliance with the Australian Code of Practice for the Care and Use of Animals and were approved by the Animal Ethics Committees of the Children, Youth and Women's Health Service and The University of Adelaide. 

Rats were randomly assigned to four groups (*n* = 10/group): Group 1: Water + Water, Group 2: Indomethacin + Water, Group 3: Indomethacin + Olive Oil, and Group 4: Indomethacin + Emu Oil. Water, Olive Oil, or Emu Oil (0.5 mL) was administered once daily (morning) via oro-gastric gavage from days 0 to 12. Between days 5 and 12, rats were administered Water (0.5 mL) or Indomethacin (Sigma-Aldrich, MO, USA; 8 mg/kg) via oro-gastric gavage (4 hours after morning gavage). 

The dose of Indomethacin (8 mg/kg) was determined from a pilot study using male Sprague Dawley rats, which was performed immediately prior to the current study. Indomethacin dosages (6 mg/kg, 8 mg/kg, and 10 mg/kg) were tested and 8 mg/kg was deemed to be the most appropriate for the current investigation, based upon metabolic parameters, disease activity index (DAI), and kill data.

#### 2.1.2. Emu Oil and Olive Oil Preparation

Commercially available Emu Oil, sourced from Emus farmed in North-Eastern South Australia, was prepared utilizing specific methodologies developed for Technology Investment Corporation by Emu Tracks (Marleston, Adelaide, South Australia). Briefly, these processes involved the rendering and filtration of Emu adipose tissue, with appropriate considerations for delivery of quality assurance and product consistency. Fatty acid analysis of Emu Oil and Olive Oil was carried out using gas chromatography, as described previously [30] (Table 1). Commercially available Olive Oil (Conga Foods, Spain) was selected as the control oil due to its high level of oleic acid; similar to that of Emu Oil. The Olive Oil control was included to determine any nonspecific oil-related effects for subsequent comparison with Emu Oil. Both Emu Oil and Olive Oil were individually stored at 4°C in 50 mL opaque containers.

### 2.2. Daily Metabolic Data and Disease Activity Index

Body weight, food and water intake, and fecal and urine output were monitored and measured daily. The severity of NSAID-induced enteropathy was assessed in a blinded fashion daily, using a DAI, which scored body weight loss, rectal bleeding, stool consistency, and overall general condition of the animal, increasing in severity on a scale of 0–3 for each parameter, which was totaled to achieve an overall DAI, as described previously [31, 32]. Overall condition was determined by (1) mobility/agility: healthy rats are considered quite active, whereas rats affected by Indomethacin characteristically become very weak and feeble, sitting hunched with very little movement and (2) fur: healthy rats are well-groomed with fur flat to the body, whereas rats administered Indomethacin become scruffy in appearance, with ruffled fur.

### 2.3. ^13^C-Sucrose Breath Test

Immediately prior to kill (day 12), the ^13^C-sucrose breath test (SBT) was performed as a noninvasive assessment of the functional status of SI health and to ensure safety of Emu Oil [33]. Briefly, following an overnight fast, a baseline breath sample was collected at *t* = 0. Rats were then gavaged with 1 mL of a 25% ^13^C-labeled sucrose solution (BDH, Merck Pty Ltd., Victoria, Australia); breath samples were collected every 15 minutes for 120 minutes, and samples were analyzed for ^13^CO_2_ concentration using an isotope ratio mass spectrometer (Europa Scientific, Crewe, UK) [34]. Data were expressed as mean percentage cumulative dose of ^13^C recovered at 90 minutes after sucrose administration (%CD90). This provides an indirect indication of the rate at which sucrose is cleaved by the enzyme sucrase in the SI, and therefore, the level of sucrase present on SI enterocytes (brush border).

### 2.4. Tissue Collection

On day 12, rats were sacrificed by CO_2_ asphyxiation followed by cervical dislocation. The GI tract was removed, measured, emptied of contents, and weighed. Furthermore, the SI tract (jejunum, jejunum-ileum junction [JI], and the ileum) was opened for intestinal damage severity assessment. Segments of the SI tract (4 cm) were collected at approximately 10% (jejunum) and 90% (ileum) of the total SI length and snap-frozen in liquid nitrogen for biochemical analysis. Samples were stored at −80°C until prepared for analysis by homogenization in 10 mM phosphate buffer. The remaining visceral organs (thymus, lungs, heart, liver, kidneys, and spleen) were weighed and discarded.

### 2.5. Intestinal Damage Severity Scoring

**Figure 1:**
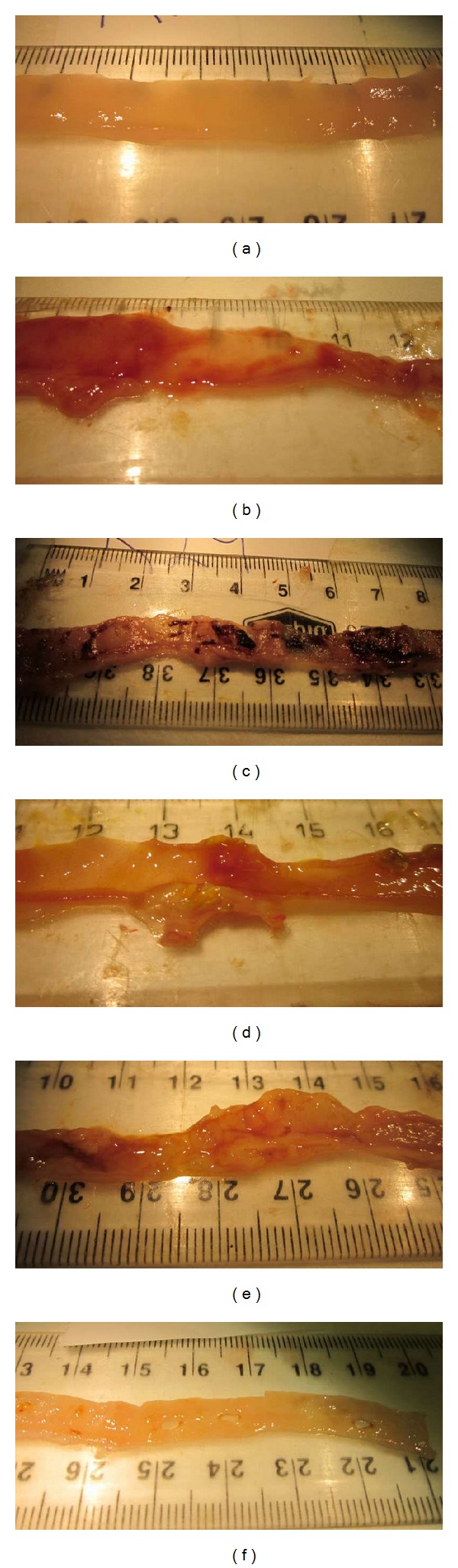
Macroscopic aspects of opened rat small intestinal sections representing (a) no damage, (b) erythema and hemorrhage, (c) severe hemorrhage, (d) large single ulcer, (e) large cluster of ulcers, and (f) perforations. These parameters were utilized in the intestinal damage severity scoring system.

Intestinal damage severity (IDS) was assessed utilizing a quantitative scoring system based on thirteen parameters. Opened intestinal sections (jejunum, JI and ileum) were inspected and quantified for erythema (small: <2 cm, large: ≥2 cm; Figure 1(b)), mild hemorrhage (small: <2 cm, large: ≥2 cm; Figure 1(b)), severe hemorrhage (small: <2 cm, large: ≥2 cm; Figure 1(c)), single ulcers (small: <0.5 cm, medium: ~0.5 cm, large: >0.5 cm; Figure 1(d)), cluster of ulcers (small: <4, large: ≥5; Figure 1(e)) and perforations (small: <0.5 cm, large: ≥0.5 cm; Figure 1(f)), in which number, size, and location were recorded on a schematic intestinal diagram during kill. Assessment was performed in a blinded fashion using a dissecting microscope (Leica Microsystems, Wetzlar, Germany). Raw parameter data were totaled to achieve an overall score for each intestinal section from each rat.

### 2.6. Biochemical Analysis

#### 2.6.1. Myeloperoxidase Activity

Myeloperoxidase (MPO) levels in the SI were determined as an indicator of neutrophil infiltration, and hence, acute inflammation, using techniques described by Howarth et al. [35]. Thawed, homogenised samples were centrifuged at 13,000 g for 12 minutes, after which the supernatant was discarded, and the tissue homogenate was resuspended in 200 *μ*L of 0.5% hexadecyltrimethyl ammonium bromide buffer, a detergent (Sigma Chemicals, Sydney, Australia). After vortexing for 2 minutes, samples were again centrifuged at 13,000 g for 2 minutes. Background, negative and positive control samples (50 *μ*L) and the supernatants of each test sample were then aliquoted into duplicate wells of a microtiter 96-well plate. Following the addition of a reaction solution (200 *μ*L to each well; 4.2 mg of O-dianisidine dihydrochloride reagent, 12.5 *μ*L H_2_O_2_, 2.5 mL potassium phosphate buffer [pH 6.0], 22.5 mL distilled water) the change in absorbance was measured at 450 nm at 1 minute intervals for 15 minutes with a spectrophotometer (Victor X4 Multilabel Reader, Perkin Elmer, Singapore). Data were expressed as units of MPO per gram of tissue.

### 2.7. Statistical Analyses

Statistical comparisons were conducted using SPSS version 16.0 for Windows (SPSS Inc., Chicago, Illinois, USA). All data sets were tested for normality of distribution using the Shapiro-Wilk statistic. Metabolic data were analysed using a one-way ANOVA and Tukey's *post hoc *test to compare groups during each period, prior to (days 0–4) and during (days 5–12) Indomethacin administration. SBT, visceral and gastrointestinal organ weights and lengths, intestinal damage severity, and MPO activity were analysed using a one-way ANOVA with Tukey's *post hoc *test. All parametric data were expressed as mean with their standard errors. DAI comparisons between groups each day were made using a Kruskal Wallis test with Mann-Whitney *U* tests and expressed as median (range). For all analyses, *P* < 0.05 was considered significant.

## 3. Results

### 3.1. Daily Metabolic Data

During the period prior to commencement of Indomethacin administration (days 0–4), Olive Oil and Emu Oil did not significantly affect body weight gain, total food and water intake, and total urine output (*P* > 0.05; Table 2) compared to normal, healthy controls. However, Olive Oil significantly reduced total fecal output in normal rats, compared to normal controls (25%; days 0–4; *P* < 0.05).

Body weight was determined as a mean percentage change from the commencement of Indomethacin or water administration (days 5–11). Water-gavaged rats gained approximately 25% body weight from day 5 to 11, whereas Indomethacin administration resulted in significantly reduced weight gain in normal rats compared to normal controls (*P* < 0.001; Table 3). Neither of the oil treatments increased body weight in Indomethacin-treated rats compared to Indomethacin-treated controls (*P* > 0.05; Table 3).

Indomethacin significantly decreased total food intake (41%) and increased water intake (72%) in normal rats compared to healthy controls during days 5–12 (*P* < 0.001; Table 3). Olive Oil and Emu Oil did not significantly affect total food or water intake in Indomethacin-treated rats compared to Indomethacin controls (*P* > 0.05). Indomethacin significantly decreased (21%; *P* < 0.01) total fecal output during days 5–12 compared to healthy controls, which was subsequently normalized following Olive Oil and Emu Oil treatment (*P* < 0.05; Table 3). Neither Indomethacin nor the oil treatments significantly affected total urine output (*P* > 0.05; Table 3).

### 3.2. Disease Activity Index (DAI)

Indomethacin significantly increased DAI in normal rats on day 10 (median score 1 (range: 0–7); *P* < 0.01), 11 (2 (1–7); *P* < 0.001) and 12 (2 (1–8); *P* < 0.001) compared to healthy controls (0 (0); Table 4). Oil treatments did not significantly affect DAI in Indomethacin-treated rats compared to Indomethacin controls (*P* > 0.05).

### 3.3. ^13^C-Sucrose Breath Test (SBT)

**Figure 2:**
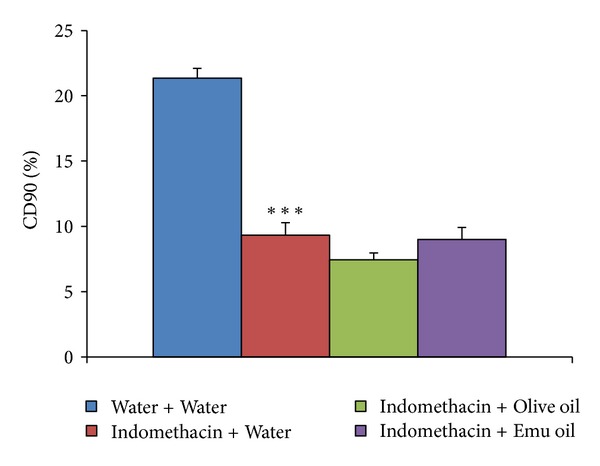
Overall functional status of small intestinal health in rats assessed utilizing the ^13^C sucrose breath test on day 12. Rats were gavaged daily with Water, Olive Oil, or Emu Oil (0.5 mL) throughout the experimental period and commenced daily gavage with water or Indomethacin (8 mg/kg) on day 5. Data are expressed as mean (% cumulative dose of ^13^C at 90 minutes; %CD90) ± standard error of the mean. *** indicates *P* < 0.001 compared to Water + Water.

Indomethacin significantly decreased percentage cumulative dose of ^13^C at 90 minutes (%CD90) compared to healthy controls (56% decrease; *P* < 0.001; Figure 2). However, both oil treatments failed to significantly increase %CD90 in Indomethacin-administered animals compared to Indomethacin controls (*P* > 0.05; Figure 2). 

### 3.4. Visceral and Gastrointestinal Organ Weights and Lengths

Organ weights were expressed as a proportion of body weight. Significant increases in duodenum (68%; *P* < 0.05), SI (48%; *P* < 0.001), caecum (48%; *P* < 0.001), and colon (24%; *P* < 0.05) weights were evident in normal rats administered Indomethacin compared to healthy controls (Table 5). Oil treatments did not significantly affect duodenum, SI, caecum, or colon weights in Indomethacin-treated rats (*P* > 0.05) compared to Indomethacin controls. There were no significant differences in visceral organ weights including heart, lungs, left kidney, right kidney, and stomach following Indomethacin or oil treatments (*P* > 0.05; Table 5).

Indomethacin significantly increased (51%) liver weight in normal rats compared to healthy controls (*P* < 0.001; Table 5). Thymus weight in Indomethacin-treated rats was decreased (55%) compared to healthy controls (*P* < 0.001; Table 5). Indomethacin significantly increased spleen weight in normal rats compared to healthy controls (88%; *P* < 0.001; Table 5). Neither Olive Oil nor Emu Oil treatment significantly affected liver, thymus, or spleen weights in rats administered Indomethacin compared to Indomethacin controls (*P* > 0.05; Table 5).

Neither Indomethacin nor oil treatments significantly affected duodenum or colon length (*P* > 0.05; Table 6). SI length in rats administered Indomethacin was significantly decreased compared to normal controls (41%; *P* < 0.001; Table 6); however, neither Emu Oil nor Olive Oil affected SI length compared to Indomethacin controls.

### 3.5. Intestinal Damage Severity (IDS) Scoring

**Figure 3:**
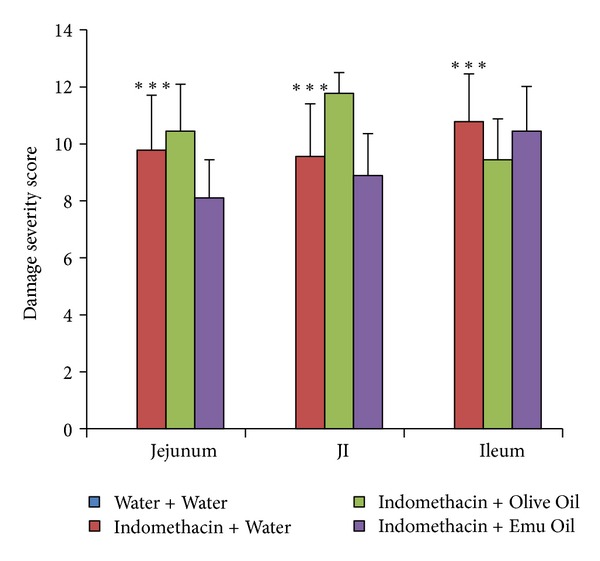
Intestinal damage severity score in the rat jejunum, jejunum-ileum junction (JI), and ileum on day 12. Rats were gavaged daily with 0.5 mL of Water, Olive Oil, or Emu Oil throughout the experimental period (days 0–12) and administered water or Indomethacin (Indo; 8 mg/kg) daily from day 5. Data are expressed as mean (severity score) ± standard error of the mean. *** indicates *P* < 0.001 compared to Water + Water.

IDS scores in the jejunum, JI, and ileum were significantly greater in rats administered Indomethacin, compared to normal controls (*P* < 0.001; Figure 3). Neither of the oil treatments decreased the overall IDS in rats administered Indomethacin, compared to Indomethacin controls (*P* > 0.05; Figure 3).

### 3.6. Myeloperoxidase (MPO) Activity

**Figure 4:**
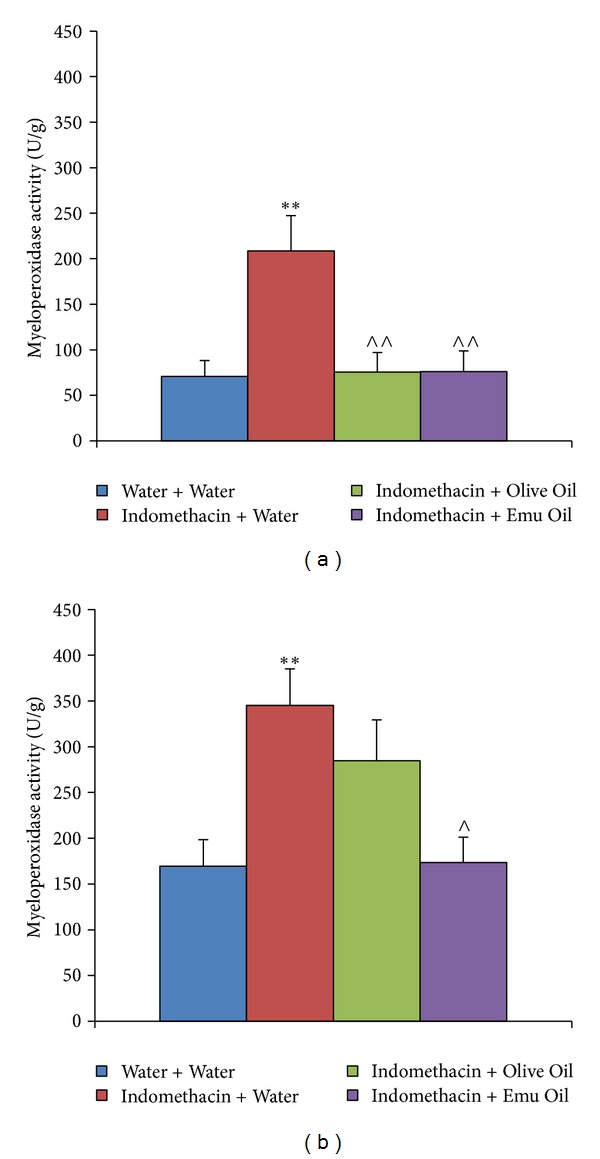
Myeloperoxidase activity indicative of acute inflammation in the rat (a) jejunum and (b) ileum on day 12. Data are expressed as mean (myeloperoxidase activity; units per gram; U/g) ± standard error of the mean. Rats were gavaged daily with 0.5 mL of Water, Olive Oil or Emu Oil throughout the experimental period (days 0–12) and administered water or Indomethacin (Indo; 8 mg/kg) daily from day 5. ** indicates *P* < 0.01 compared to Water + Water;  ^∧∧^ indicates *P* < 0.01,  ^∧^ indicates *P* < 0.05 compared to Indomethacin + Water.

Jejunal MPO activity, indicative of acute inflammation, was increased by 195% following Indomethacin administration, compared to healthy controls (*P* < 0.01; Figure 4(a)). In the jejunum, both Olive Oil and Emu Oil significantly decreased MPO activity in Indomethacin-treated rats compared to Indomethacin controls (64%; *P* < 0.01). Ileal MPO activity was significantly elevated in normal rats administered Indomethacin compared to healthy controls (104%; *P* < 0.01; Figure 4(b)). However, in the ileum, only Emu Oil significantly reduced MPO activity in Indomethacin-administered rats compared to Indomethacin controls (50%; *P* < 0.05; Figure 4(b)). Olive Oil did not significantly affect ileal MPO activity (*P* > 0.05).

## 4. Discussion

NSAIDs have been widely used to achieve analgesic, antinociceptive, and anti-inflammatory effects; however, a major limitation of NSAID usage has been related to GI side effects. Emu Oil has been reported to reduce the severity of experimentally induced inflammatory GI disorders including IBD [24] and chemotherapy-induced intestinal mucositis [28]. The current study indicated that Emu Oil reduced SI neutrophil infiltration and normalized total fecal output in a rat model of Indomethacin-induced enteropathy. Furthermore, Emu Oil treatment significantly increased liver and spleen weight and decreased thymus weight relative to Indomethacin controls. However, Emu Oil treatment failed to improve most other parameters associated with Indomethacin-induced SI damage.

In the current study, an 8 mg/kg Indomethacin dose was employed, resulting in clinical manifestations which included significantly decreased food intake accompanied by weight loss and reduced fecal output and increased water intake, with no changes in urine output. Furthermore, elevated DAI, reduced brush border sucrase activity, as assessed indirectly by the SBT and elevated IDS were observed. In a study by Kamil et al. [2], rats were administered Indomethacin daily at 6 mg/kg for 7 days to induce enteropathy. Interestingly, no clinical signs of enteropathy were observed, highlighting the clinical significance of minor dosage increases of Indomethacin. Clinical manifestations of Indomethacin-enteropathy were not improved by Olive Oil or Emu Oil treatment. Moreover, fecal output was increased following oil treatment relative to Indomethacin controls, consistent with the study by Abimosleh et al. [24] in a rat model of IBD. This may have represented a normalization of fecal output compared to Indomethacin controls. Alternatively, increased water intake coincident with the absence of changes in urine output may have indicated water loss in stools and subsequent dehydration. 

In the current study, Indomethacin decreased SI length, a phenomenon reported to be a compensatory mechanism in response to intestinal damage to increase mucosal surface area [36] and potentially protect the intestine from pathogenic invasion. Furthermore, Indomethacin increased duodenum, SI, and colonic weight. Although not quantified, these weight increases may have represented mucosal, submucosal or muscularis hyperemia and oedema, potentially combined with inflammatory cell infiltration and the associated exudate. This would likely cause an increase in GI weight. Emu Oil did not improve Indomethacin-enteropathy manifestations associated with intestinal weights. 

Recent studies have hypothesized that neutrophil activation is an important primary event preceding Indomethacin-induced SI injury [37]. Fukumoto et al. [10] demonstrated that tissue-associated neutrophil accumulation, assessed by MPO activity, and keratinocyte chemoattractant (KC) mRNA expression, involved in chemotaxis and neutrophil activation, were significantly increased in Indomethacin-treated SI mucosa [10]. Furthermore, MPO levels and KC mRNA expression were reduced in TNF-*α*-deficient mice treated with Indomethacin compared to wild-type. This indicated a protective role of TNF-*α* deficiency in decreasing Indomethacin-induced SI mucosal injury [10]. In the current study, Indomethacin increased MPO activity in the jejunum and ileum. Both Olive Oil and Emu Oil treatment significantly decreased MPO activity, indicative of neutrophil infiltration, in the Indomethacin-damaged jejunum. Importantly, only Emu Oil decreased MPO levels in the ileum, relative to Indomethacin controls. Emu Oil has previously been shown to reduce levels of TNF-*α* in a croton-oil induced model of auricular swelling [27]. Therefore, the observed decrease in MPO levels following Emu Oil administration in the current study may have been the result of suppressed TNF-*α* expression.

PUFAs influence cytokine production, lymphocyte proliferation, and macrophage activation state through modulation of AA (common n-6 PUFA) metabolism [38]. n-3 PUFAs replace AA from phospholipid membranes, thereby reducing the availability of AA to COX and thus reducing the production of PGs involved in the inflammatory cascade such as the 2-series eicosanoids. Furthermore, n-3 PUFAs can be directly metabolized by COX, resulting in the production of anti-inflammatory 3-series eicosanoids [38]. Cell culture studies with n-3 PUFAs eicosapentanoic acid (EPA) and docosahexaenoic acid (DHA) have been shown to inhibit lipopolysaccharide-induced production of COX-2, and proinflammatory cytokines including TNF-*α*, IL-1, IL-6, IL-8, and IL-12 in endothelial cells [19, 20]. Furthermore, animal feeding studies with Fish Oil, a source of EPA and DHA, supported *in vitro* observations of decreased cytokine production [22, 39, 40]. Further studies of Emu Oil could benefit from a complete time-course of cytokine profiling to identify potential effects on proinflammatory cytokine antagonism.

Liver weight was significantly increased in the current study following Indomethacin (8 mg/kg), compared to normal controls. In a previous rat model of Indomethacin-enteropathy, daily administration of Indomethacin at 6 mg/kg did not result in significant liver weight changes. The impact of Indomethacin on liver weight therefore appears to be dose-dependent as Ilic et al. [41], consistent with the current findings, demonstrated liver weight increases in male albino Wistar rats injected daily for three days with 12.5 mg/kg of diclofenac (NSAID). The authors observed pronounced parenchymal necrosis, elevated eosinophilic hepatocytes, extensive microvesicular steatosis, and sinusoidal dilation [41]. Moreover, TNF-*α*, directly linked to the pathogenesis of enteropathy, binds to TNF-RI cell death receptors leading to hepatocyte apoptosis followed by accumulation of neutrophils [42]. Future studies would benefit from liver enzyme analyses including gamma-glutamyl transpeptidase (GGT), alanine aminotransaminase (ALT), alkaline phosphotase (ALP), and bilirubin, which elevate in NSAID-induced toxicity [43]. Furthermore, histological assessment of Kupffer cells, resident liver macrophages [44], hepatotoxic lymphocytes including CD4^+^, CD8^+^ T cells and natural killer cells and apoptotic hepatocytes [45] would provide greater insight into the hepatic impact of Indomethacin.

In the current study, Olive Oil and Emu Oil treatment to rats which were administered Indomethacin did not significantly affect liver weight, relative to Indomethacin controls. Abimosleh et al. [24] examined liver weight in healthy Sprague Dawley rats, which was not significantly affected by Olive Oil or Emu Oil. This indicated that the current findings were directly related to Indomethacin administration with no effect of oil administration. Valenzuela et al. [46] demonstrated that n-3 long-chain PUFA supplementation induced an antioxidant response preventing liver steatosis in mice fed a high fat diet. This was further supported by a study in which Fish Oil in combination with Indomethacin decreased the severity of liver steatosis in a mouse model of familial hypercholesterolemia [38]. Based upon the n-3 PUFAs present in Emu Oil and its reported antioxidant activity [47], liver steatosis seems less likely in the current study. However, a high n-6/n-3 PUFA ratio has been implicated in nonalcoholic FA liver disease [48] and therefore it would be beneficial to determine the effects of FA ratio and n-9 FA levels on liver function in future studies.

Thymus weight was significantly decreased following administration of 8 mg/kg Indomethacin in the current study, relative to normal controls. Kamil et al. [2] did not report any thymus weight changes using 6 mg/kg of oral Indomethacin. However, a single subcutaneous injection of Indomethacin at 35 mg/kg significantly decreased thymus weight in rats after three days in a study by Filaretova et al. [36], consistent with the current study. Furthermore, the increased spleen weight evident following Indomethacin administration in the current study should be pursued in relation to the thymus weight decreases. Such studies could determine the contribution of T (CD4^+^ and CD8^+^) and B lymphocyte upregulation or suppression in the thymus and spleen, respectively. However, neither Olive Oil nor Emu Oil significantly affected thymus or spleen weight. 

## 5. Conclusions

In summary, when administered once daily to rats, Emu Oil significantly reduced acute inflammatory activity associated with Indomethacin-induced enteropathy. However, although encouraging, it appears likely that increased dose and/or frequency of administration will need to be explored in order to achieve demonstrable clinical benefit in the setting of NSAID-enteropathy.

## Figures and Tables

**Table 1:** Major fatty acid (FA) composition of Emu and Olive Oils used in the current study including unsaturated fatty acid (UFA) and saturated fatty acid (SFA) ratios. Monounsaturated fatty acid (MUFA); polyunsaturated fatty acid (PUFA).

Major FA composition of Emu and Olive Oils			
Fatty acid	Common name	Emu	Olive
16 : 0	Palmitic acid	24	10.4
16 : 1n-7	Palmitoleic acid	4.3	0.7
18 : 0	Stearic acid	8.5	3.1
18 : 1n-9	Oleic acid	49.1	73.9
18 : 2n-6	Linoleic acid	9.5	8.4
18 : 3n-3	*α*-Linolenic acid	1.1	0.7
Saturated		32.5	13.5
MUFA		53.4	74.6
PUFA		10.6	9.1
UFA: SFA ratio		2	6.2

**Table 2:** Body weight (% change from starting body weight), total food (g) and water (mL) intake and total fecal (g) and urine (mL) output in normal rats during the period prior to Indomethacin administration (days 0–4). Rats were gavaged daily with Water, Olive Oil, or Emu Oil (0.5 mL).

	Water	Olive Oil	Emu Oil
Body weight (%)	14.8 ± 0.5	14.8 ± 0.6	14.4 ± 0.6
Food intake (g)	85.1 ± 1.5	83.4 ± 1.9	84.6 ± 2
Water intake (mL)	353.6 ± 38.9	429.4 ± 50.6	425.6 ± 58.6
Fecal ouput (g)	7.8 ± 0.4	6.1 ± 0.5*	7.1 ± 0.3
Urine ouput (mL)	65.2 ± 3.6	66.9 ± 6.5	68.2 ± 4.2

*P < 0.05 compared to Water group.

Data are expressed as mean (%, g or mL) ± standard error of the mean.

**Table 3:** Body weight (% change from starting body weight), total food (g) and water (mL) intake and total fecal (g) and urine (mL) output in rats during Indomethacin administration (days 5–12). Rats were gavaged daily with Water, Olive Oil, or Emu Oil (0.5 mL) throughout the experimental period and commenced daily gavage with water or Indomethacin (Indo; 8 mg/kg) on day 5. Rats were fasted overnight on day 11 and therefore day 12 body weight data was not included.

	Water + Water	Indo + Water	Indo + Olive Oil	Indo + Emu Oil
Body weight (%)	23.1 ± 0.8	1.6 ± 3.3***	6.0 ± 2.4	4.6 ± 2.0
Food intake (g)	132.8 ± 1.6	78.2 ± 4.9***	83.9 ± 4.3	77.9 ± 4.4
Water intake (mL)	416 ± 51.3	715.1 ± 72.1**	651.7 ± 53.1	655.9 ± 66.1
Fecal ouput (g)	12.8 ± 0.4	10.1 ± 0.6**	12.4 ± 0.5^∧^	12.4 ± 0.7^∧^
Urine ouput (mL)	104.2 ± 8.5	87.8 ± 3.1	85.4 ± 6.2	98.8 ± 7.3

Data are expressed as mean (%, g or mL) ± standard error of the mean. ***P < 0.001, **P < 0.01 compared to Water + Water;  ^∧^ *P* < 0.05 compared to Indomethacin + Water.

**Table 4:** Disease activity index (DAI) of rats gavaged daily with 0.5 mL of Water, Olive Oil, or Emu Oil throughout the experimental period (days 0–12) and administered water or Indomethacin (Indo; 8 mg/kg) daily on day 5. Data are expressed as median disease activity index score (range).

Day of trial	Water + Water	Indo + Water	Indo + Olive Oil	Indo + Emu Oil
6	0 (0)	0 (0)	0 (0)	0 (0)
7	0 (0)	0 (0)	0 (0)	0 (0)
8	0 (0)	0 (0–2)	0 (0)	0 (0)
9	0 (0)	0 (0–6)	0 (0-1)	0 (0–3)
10	0 (0)	1 (0–7)**	1 (0–3)	0 (0–4)
11	0 (0)	2 (1–7)***	2 (0–4)	1 (0–5)
12	0 (0)	2 (1–8)***	3 (0–5)	2 (0–7)

***P < 0.001, **P < 0.01 compared to Water + Water.

**Table 5:** Organ weight following adjustment for rat body weight. Rats were gavaged daily with 0.5 mL of Water, Olive Oil, or Emu Oil throughout the experimental period (days 0–12) and administered water or Indomethacin (Indo; 8 mg/kg) daily from day 5.

Weight (%)	Water + Water	Indo + Water	Indo + Olive Oil	Indo + Emu Oil
Thymus	27 ± 1	12 ± 3***	13 ± 2	14 ± 2
Heart	42 ± 0	41 ± 2	42 ± 2	40 ± 1
Lungs	62 ± 3	59 ± 4	61 ± 4	57 ± 3
L Kidney	40 ± 1	43 ± 1	41 ± 1	41 ± 2
R Kidney	44 ± 4	42 ± 1	52 ± 6	42 ± 2
Liver	352 ± 9	533 ± 23***	525 ± 25	537 ± 29
Spleen	30 ± 1	56 ± 5***	58 ± 3	64 ± 5
Stomach	56 ± 2	66 ± 7	59 ± 11	57 ± 4
Duodenum	22 ± 1	37 ± 5*	49 ± 5	39 ± 6
SI	178 ± 5	263 ± 38***	330 ± 17	270 ± 40
Caecum	29 ± 1	43 ± 2***	46 ± 3	46 ± 3
Colon	38 ± 1	47 ± 1*	50 ± 2	49 ± 3

Data are expressed as mean (% relative to body weight) ± standard error of the mean.

All values ×10^−2^. ***P < 0.001, *P < 0.05 compared to Water + Water.

**Table 6:** Gastrointestinal organ length of rats gavaged daily with 0.5 mL of Water, Olive Oil, or Emu Oil throughout the experimental period (days 0–12) and administered water or Indomethacin (Indo; 8 mg/kg) daily from day 5.

Length (cm)	Water + Water	Indo + Water	Indo + Olive Oil	Indo + Emu Oil
Duodenum	6.5 ± 0.3	5.6 ± 0.3	6.6 ± 0.4	6.2 ± 0.4
SI	78.5 ± 2.2	46.2 ± 2.1***	51.0 ± 3.4	51.0 ± 3.4
Colon	12.9 ± 0.4	11.8 ± 0.6	12.0 ± 0.4	12.2 ± 0.5

Data are expressed as mean (cm) ± standard error of the mean.

***P < 0.001 compared to Water + Water.

## References

[1] Green GA (2001). Understanding NSAIDs: from aspirin to COX-2. *Clinical Cornerstone*.

[2] Kamil R, Geier MS, Butler RN, Howarth GS (2007). Lactobacillus rhamnosus GG exacerbates intestinal ulceration in a model of indomethacin-induced enteropathy. *Digestive Diseases and Sciences*.

[3] Scarpignato C (2008). NSAID-induced intestinal damage: are luminal bacteria the therapeutic target?. *Gut*.

[4] Hawcroft G, D'Amico M, Albanese C, Markham AF, Pestell RG, Hull MA (2002). Indomethacin induces differential expression of *β*-catenin, *γ*-catenin and T-cell factor target genes in human colorectal cancer cells. *Carcinogenesis*.

[5] Boelsterli UA, Redinbo MR, Saitta KS (2012). Multiple NSAID-induced hits injure the small intestine: underlying mechanisms and novel strategies. *Toxicological Sciences*.

[6] Scarpignato C, Hunt RH (2010). Nonsteroidal antiinflammatory drug-related injury to the gastrointestinal tract: clinical picture, pathogenesis, and prevention. *Gastroenterology Clinics of North America*.

[7] Reuter BK, Davies NM, Wallace JL (1997). Nonsteroidal anti-inflammatory drug enteropathy in rats: role of permeability, bacteria, and enterohepatic circulation. *Gastroenterology*.

[8] Somasundaram S, Sigthorsson G, Simpson RJ (2000). Uncoupling of intestinal mitochondrial oxidative phosphorylation and inhibition of cyclooxygenase are required for the development of NSAID-enteropathy in the rat. *Alimentary Pharmacology and Therapeutics*.

[9] Lanas A, Scarpignato C (2006). Microbial flora in NSAID-induced intestinal damage: a role for antibiotics?. *Digestion*.

[10] Fukumoto K, Naito Y, Takagi T (2011). Role of tumor necrosis factor-*α* in the pathogenesis of indomethacin-induced small intestinal injury in mice. *International Journal of Molecular Medicine*.

[11] Takeuchi K, Tanigami M, Amagase K, Ochi A, Okuda S, Hatazawa R (2010). Endogenous prostaglandin E2 accelerates healing of indomethacin-induced small intestinal lesions through upregulation of vascular endothelial growth factor expression by activation of EP4 receptors. *Journal of Gastroenterology and Hepatology*.

[12] Takeuchi K, Tanaka A, Kato S, Amagase K, Satoh H (2010). Roles of COX inhibition in pathogenesis of NSAID-induced small intestinal damage. *Clinica Chimica Acta*.

[13] Patrignani P, Tacconelli S, Sciulli MG, Capone ML (2005). New insights into COX-2 biology and inhibition. *Brain Research Reviews*.

[14] Sigthorsson G, Simpson RJ, Walley M (2002). COX-1 and 2, intestinal integrity, and pathogenesis of nonsteroidal anti-inflammatory drug enteropathy in mice. *Gastroenterology*.

[15] Maehata Y, Esaki M, Morishita T (2012). . Small bowel injury induced by selective cyclooxygenase-2 inhibitors: a prospective, double-blind, randomized clinical trial comparing celecoxib and meloxicam. *Journal of Gastroenterology*.

[16] Maiden L, Thjodleifsson B, Seigal A (2007). Long-term effects of nonsteroidal anti-inflammatory drugs and cyclooxygenase-2 selective agents on the small bowel: a cross-sectional capsule enteroscopy study. *Clinical Gastroenterology and Hepatology*.

[17] Miles EA, Calder PC (2012). Influence of marine *n* − 3 polyunsaturated fatty acids on immune function and a systematic review of their effects on clinical outcomes in rheumatoid arthritis. *The British Journal of Nutrition*.

[18] Tenikoff D, Murphy KJ, Le M, Howe PR, Howarth GS (2005). Lyprinol (stabilised lipid extract of New Zealand green-lipped mussel): a potential preventative treatment modality for inflammatory bowel disease. *Journal of Gastroenterology*.

[19] Lo CJ, Chiu KC, Fu M, Lo R, Helton S (1999). Fish oil decreases macrophage tumor necrosis factor gene transcription by altering the NF*κ*B activity. *Journal of Surgical Research*.

[20] Khalfoun B, Thibault F, Watier H, Bardos P, Lebranchu Y (1997). Docosahexaenoic and eicosapentaenoic acids inhibit in vitro human endothelial cell production of interleukin-6. *Advances in Experimental Medicine and Biology*.

[21] De Caterina R, Libby P (1996). Control of endothelial leukocyte adhesion molecules by fatty acids. *Lipids*.

[22] Whiting CV, Bland PW, Tarlton JF (2005). Dietary *n* − 3 polyunsaturated fatty acids reduce disease and colonic proinflammatory cytokines in a mouse model of colitis. *Inflammatory Bowel Diseases*.

[23] Abimosleh SM, Tran CD, Howarth GS (2012). Emu oil: a novel therapeutic for disorders of the gastrointestinal tract?. *Journal of Gastroenterology and Hepatology*.

[24] Abimosleh SM, Lindsay RJ, Butler RN (2012). Emu oil increases colonic crypt depth in a rat model of ulcerative colitis. *Digestive Diseases and Sciences*.

[25] Whitehouse MW, Turner AG, Davis CKC, Roberts MS (1998). Emu oil(s): a source of non-toxic transdermal anti-inflammatory agents in aboriginal medicine. *Inflammopharmacology*.

[26] Snowden JM, Whitehouse MW (1997). Anti-inflammatory activity of emu oils in rats. *Inflammopharmacology*.

[27] Yoganathan S, Nicolosi R, Wilson T (2003). Antagonism of croton oil inflammation by topical emu oil in CD-1 mice. *Lipids*.

[28] Lindsay RJ, Geier MS, Yazbeck R, Butler RN, Howarth GS (2010). Orally administered emu oil decreases acute inflammation and alters selected small intestinal parameters in a rat model of mucositis. *British Journal of Nutrition*.

[29] Tomas FM, Knowles SE, Owens PC (1991). Effects of full-length and truncated insulin-like growth factor-I on nitrogen balance and muscle protein metabolism in nitrogen-restricted rats. *Journal of Endocrinology*.

[30] Portolesi R, Powell BC, Gibson RA (2007). Competition between 24:5*n* − 3 and ALA for Δ6 desaturase may limit the accumulation of DHA in HepG2 cell membranes. *Journal of Lipid Research*.

[31] Murthy SNS, Cooper HS, Shim H, Shah RS, Ibrahim SA, Sedergran DJ (1993). Treatment of dextran sulfate sodium-induced murine colitis by intracolonic cyclosporin. *Digestive Diseases and Sciences*.

[32] Howarth GS, Xian CJ, Read LC (2000). Predisposition to colonic dysplasia is unaffected by continuous administration of insulin-like growth factor-I for twenty weeks in a rat model of chronic inflammatory bowel disease. *Growth Factors*.

[33] Pelton NS, Tivey DR, Howarth GS, Davidson GP, Butler RN (2004). A novel breath test for the non-invasive assessment of small intestinal mucosal injury following methotrexate administration in the rat. *Scandinavian Journal of Gastroenterology*.

[34] Tooley KL, Howarth GS, Butler RN (2009). Mucositis and non-invasive markers of small intestinal function. *Cancer Biology and Therapy*.

[35] Howarth GS, Francis GL, Cool JC, Xu X, Byard RW, Read LC (1996). Milk growth factors enriched from cheese whey ameliorate intestinal damage by methotrexate when administered orally to rats. *Journal of Nutrition*.

[36] Filaretova LP, Bagaeva TR, Morozova OY (2011). The healing of NSAID-induced gastric lesion may be followed by small intestinal and cardiovascular side effects. *Journal of Physiology and Pharmacology*.

[37] Santucci L, Fiorucci S, Di Matteo FM, Morelli A (1995). Role of tumor necrosis factor *α* release and leukocyte margination in indomethacin-induced gastric injury in rats. *Gastroenterology*.

[38] Murali G, Milne GL, Webb CD (2012). Fish oil and indomethacin in combination potently reduce dyslipidemia and hepatic steatosis in LDLR ^−/−^ mice. *The Journal of Lipid Research*.

[39] Renier G, Skamene E, DeSanctis J, Radzioch D (1993). Dietary *n* − 3 polyunsaturated fatty acids prevent the development of atherosclerotic lesions in mice: modulation of macrophage secretory activities. *Arteriosclerosis and Thrombosis*.

[40] Yaqoob P, Calder P (1995). Effects of dietary lipid manipulation upon inflammatory mediator production by murine macrophages. *Cellular Immunology*.

[41] Ilic S, Drmic D, Franjic S (2011). Pentadecapeptide BPC 157 and its effects on a NSAID toxicity model: diclofenac-induced gastrointestinal, liver, and encephalopathy lesions. *Life Sciences*.

[42] Mouzaoui S, Rahim I, Djerdjouri B (2012). Aminoguanidine and curcumin attenuated tumor necrosis factor (TNF)-*α*-induced oxidative stress, colitis and hepatotoxicity in mice. *International Immunopharmacology*.

[43] Herath CB, Mak K, Burrell LM (2012). Angiotensin-(1-7) reduces the portal pressure response to angiotensin II and methoxamine via an endothelial nitric oxide mediated pathway in cirrhotic rat liver. *American Journal of Physiology Gastrointestinal and liver Physiology*.

[44] Abdel-Zaher AO, Abdel-Rahman MM, Hafez MM, Omran FM (2007). Role of nitric oxide and reduced glutathione in the protective effects of aminoguanidine, gadolinium chloride and oleanolic acid against acetaminophen-induced hepatic and renal damage. *Toxicology*.

[45] Wakabayashi H, Ito T, Fushimi S (2012). Spred-2 deficiency exacerbates acetaminophen-induced hepatotoxicity in mice. *Clinical Immunology*.

[46] Valenzuela R, Espinosa A, Gonzalez-Manan D (2012). *N* − 3 long-chain polyunsaturated fatty acid supplementation significantly reduces liver oxidative stress in high fat induced steatosis. *PLoS ONE*.

[47] Bennett DC, Code WE, Godin DV, Cheng KM (2008). Comparison of the antioxidant properties of emu oil with other avian oils. *Australian Journal of Experimental Agriculture*.

[48] Araya J, Rodrigo R, Videla LA (2004). Increase in long-chain polyunsaturated fatty acid *n* − 6/*n* − 3 ratio in relation to hepatic steatosis in patients with non-alcoholic fatty liver disease. *Clinical Science*.

